# A decline in female baboon hypothalamo-pituitary-adrenal axis activity anticipates aging

**DOI:** 10.18632/aging.101235

**Published:** 2017-05-09

**Authors:** Shanshan Yang, Kenneth G. Gerow, Hillary F. Huber, McKenna M. Considine, Cun Li, Vicki Mattern, Anthony G. Comuzzie, Stephen P. Ford, Peter W. Nathanielsz

**Affiliations:** 1 Texas Biomedical Research Institute, San Antonio, TX 78227, USA; 2 Department of Neurology, the First Affiliated Hospital of Harbin Medical University, Harbin, Heilongjiang 150081, China; 3 Texas Pregnancy and Life-course Health Research Center, Department of Animal Sciences, University of Wyoming, Laramie, WY 82071, USA; 4 Department of Statistics, University of Wyoming, Laramie, WY 82071, USA; 5 Department of Animal Sciences, University of Wyoming, Laramie, WY 82071, USA

**Keywords:** baboon, glucocorticoids, paraventricular nucleus, HPA axis (hypothalamus-pituitary-adrenal), aging

## Abstract

Stressors that disrupt homeostasis advance aging. Glucocorticoids regulate multiple processes that determine the aging trajectory. Debate exists regarding life-course circulating glucocorticoid concentrations. Rodent and nonhuman primate studies indicate circulating glucocorticoids fall from early life. We measured fasting morning cortisol in 24 female baboons (6-21 years, human equivalent ~18-70). We also quantified hypothalamic paraventricular nuclear (PVN) arginine vasopressin (AVP), corticotropin-releasing hormone, steroid receptors, and pituitary proopiomelanocortin immunohistochemically in 14 of these females at 6-13 years. We identified significant age-related 1) linear fall in cortisol and PVN AVP from as early as 6 years; 2) increased PVN glucocorticoid and mineralocorticoid receptors; 3) increased PVN 11β-hydroxysteroid dehydrogenase 1 and 2, regulators of local cortisol production, and 4) decreased pituitary proopiomelanocortin. Our data identify increased age-related negative feedback and local PVN cortisol production as potential mechanisms decreasing PVN drive to hypothalamo-pituitary-adrenal axis activity that result in the age-related circulating cortisol fall. Further studies are needed to determine whether the cortisol fall 1) causes aging, 2) protects by slowing aging, or 3) is an epiphenomenon unrelated to aging processes. We conclude that aging processes are best studied by linear life-course analysis beginning early in life.

## INTRODUCTION

Of the roughly 150,000 people who die daily in the world, about two thirds die of age-related causes [1]. In industrialized nations, the proportion is much higher, reaching 90% [2]. There are three main general theories of aging: genetic mutation, wear and tear (both proposed in the 1950s by Denham Harman [3]), and accumulated functional impairments in multiple tissues in the body [4, 5]. Each of these mechanisms almost certainly plays a part, as aging is most likely a multifactorial process in which gene-environment interactions play a central role.

There is much controversy regarding the profile of the trajectory of circulating glucocorticoids across the life-course (see [6]). There are pronounced differences in the circulating profiles of glucocorticoids between rodents and primates in fetal and early life [7] and it remains to be determined whether these marked differences early in life have sequelae in later life including differences in aging glucocorticoid profiles. In the rat we have demonstrated a fall in circulating corticosterone between postnatal days (PND) 450 and 650 [6]. Elucidation of changes in cortisol in aging is best achieved by commencing measurements of cortisol concentrations even before a clear aged phenotype emerges. We propose that the best method of analysis of age-related changes in hormone and other functions is by linear regression with adequate samples at multiple time points across the life-course period studied, rather than studies of discrete, age-related groups spaced at different stages in the life-course and analyzed with traditional statistics such as *t*-test and ANOVA [6]. In addition most studies are based across relatively small age windows of data making it difficult to draw conclusions on events across the periods lacking data between the documented stages. This is especially true if there are long time gaps between the data sets.

One recent carefully controlled baboon study of age related changes in cortisol in blood provides support to our rodent findings. This baboon study used regression analysis across the life-course in samples from 33 females and 5 males from 6–26 years of age (mean 14 years), and reported a linear decrease in cortisol from the youngest age studied [8]. Although a linear correlation cannot be made between baboon and human years, this baboon age range would correspond approximately to a human age range of 18–78 years [9, 10]. The average lifespan of baboons in captivity has been reported as approximately 21 years [11].

Our approach to investigations within our baboon (*Papio hamadryas*) colony is to conduct many of the studies in group social housing with full physical activity. In this situation it is necessary to tranquilize baboons to obtain blood samples. Thus we first determined that the dose of ketamine necessary to obtain a femoral venous blood sample does not elevate blood cortisol within the time required to obtain the sample. We then sought to confirm the recently reported gradual linear decline in circulating cortisol from a relatively early stage in the baboon life-course [8] by measuring circulating cortisol in healthy female baboons from 6–21 years of age. Having confirmed the fall, we sought evidence for changes in hypothalamic paraventricular nucleus (PVN) regulators of hypothalamo-pituitary-adrenal axis (HPAA) function from 6-13 years. We hypothesized that commensurate with the cortisol fall there would be evidence of increased negative feedback on the HPAA indicated by increased glucocorticoid (GR) and mineralocorticoid (MR) receptor, resulting in decreased PVN arginine vasopressin (AVP) and corticotropin-releasing hormone (CRH) and decreased pituitary pro-opiomelanocortin (POMC).

In addition to production by the adrenal cortex, cortisol is produced in multiple peripheral tissues by reduction of cortisone to cortisol by 11beta hydroxysteroid dehydrogenase Type 1 (11β-HSD1). This conversion is reversed by 11β-HSD2. Thus locally produced cortisol increases or decreases according to the balance of these two reactions as we have shown in fetal baboons [12]. A functional local role for these enzymes and their co-factors in regulating local cortisol activity is supported by the demonstration that intra-hippocampal glucocorticoids generated by 11β-HSD1 affect memory in aged mice [13]. To determine if local cortisol production might also play a role we sought to determine the abundance of these local factors in the PVN as evidence of altered local generation of cortisol via the 11βHSD system.

Objectives: 1) Measure fasting morning cortisol in 24 female baboons across more than 50% of the life-course, from 6-21 years of age, to confirm the life-course fall in cortisol. 2) Quantify hypothalamic PVN and pituitary proteins that regulate HPAA function in 14 female baboons, 6-13 years of age. 3) Test for associations between age and proteins of the hypothalamus and pituitary to identify the major drive to basal HPAA function from early adult life through early aging.

## RESULTS

### Evidence that cortisol concentration in baboon peripheral blood is not altered within 10 minutes of ketamine administration

**Figure 1:**
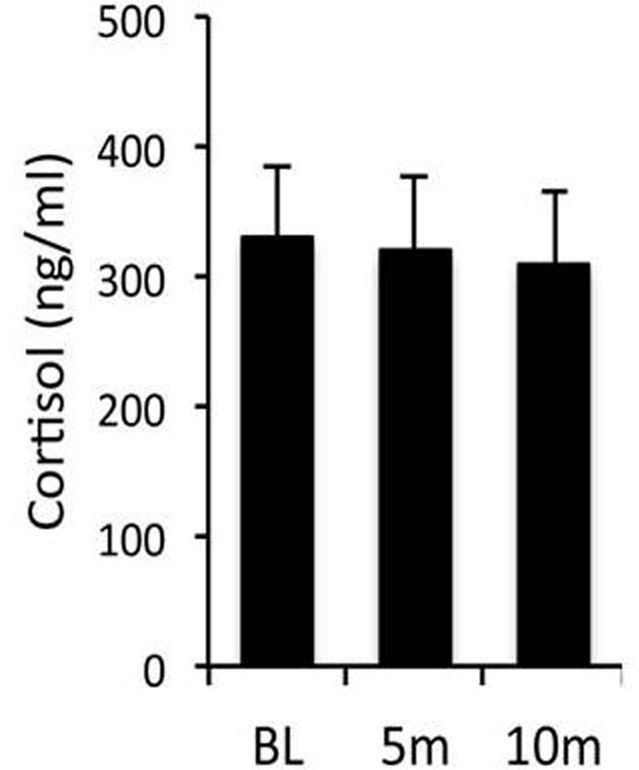
Pilot study showing effect of ketamine timing on cortisol Study was conducted on fasting baboons (*P. hamadryas*) between 08.00 and 09.00 am to evaluate acute effect within 10 minutes (m) of injection of ketamine (10mg/kg IM) on serum cortisol. Baseline (BL) cortisol in venous samples removed when baboons are fully conscious and free moving on a tether system without administration of any agents in the last 24 h. Ketamine administered and samples taken at 5 and 10 min after ketamine (n = 12; 6 male and 6 female; Mean + SEM).

Figure 1 shows that serum cortisol values do not increase from basal within 10 minutes of ketamine administration, indicating that cortisol values obtained within 10 min of tranquilisation with ketamine in the outdoor cage situation were basal.

### Age related fall in circulating cortisol

**Figure 2:**
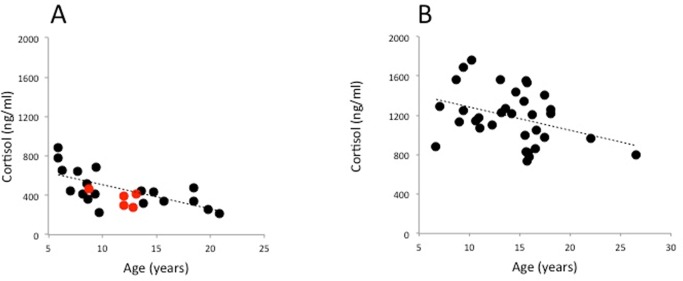
Female baboon (*P. hamadryas*) linear regression of serum cortisol by age (**A**) 24 baboons from the colony at Southwest National Primate Research Center (cortisol = −24.7*X+735.1 ng/ml; P=0.0007); (**B**) 31 baboons from the Oklahoma University Primate Center (colony cortisol = −23.7*X+1521; P=0.039). The data for the Oklahoma baboons are reproduced from their publication (Willis et al. 2014). Red symbols represent five baboons that were included in the immunohistochemistry studies reported here.

Serum cortisol concentration showed a strong negative correlation with baboon age. It is of interest that the annual fall in our study (24.7 ng.ml ^−1^.y^—1^) was almost identical to that in the Oklahoma study (23.7 ng.ml ^−1^.y^−−1^), presented here with permission (Fig. 2).

### Abundance and age-related correlations of PVN immunoreactive regulatory proteins

**Figure 3:**
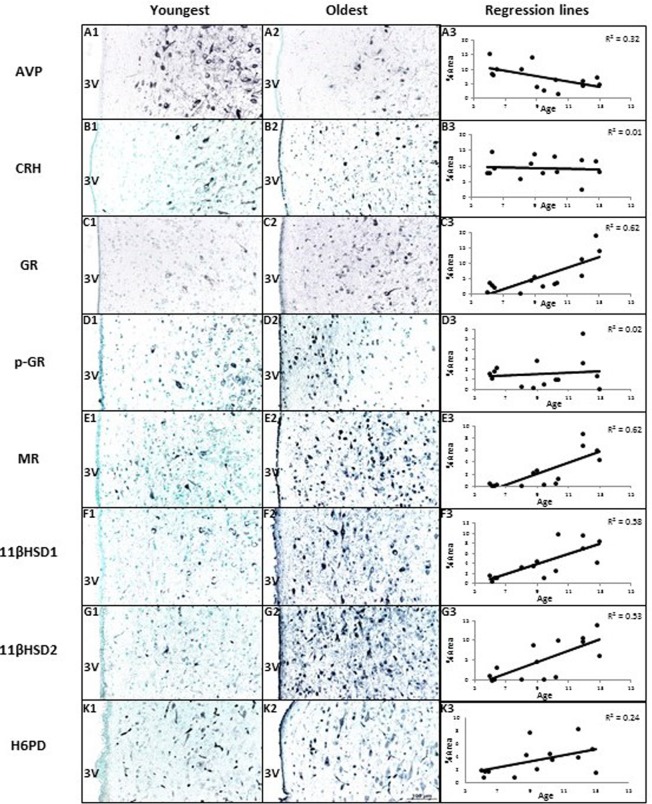
The effects of age on area% positive staining of HPAA proteins in hypothalamus of female baboons (*P. hamadryas*) Columns A1-K1 and A2-K2 show the youngest and oldest photomicrographs. Column A3-K3 shows correlations between age and peptides of hypothalamus. Linear regression showed AVP expression was negatively correlated with age (R = −0.57, *P* < 0.05). GR, MR, 11βHSD1, and 11βHSD2 showed positive age-related regression in the PVN of hypothalamus (R values were as follows: 0.78, 0.79, 0.76, 0.73, *P* < 0.05, respectively). CRH and p-GR were not correlated with ages (*P* > 0.05). The expression of H6PD tended to correlate positively with age in the PVN of hypothalamus (R = 0.49, *P* = 0.08). Scale bar (100µm) applies to all micrographs.

AVP, CRH, GR, p-GR, MR, 11βHSD1, 11βHSD2, and H6PD were all detected in the PVN. Figure 3 shows AVP expression was negatively correlated with age (R = −0.57, *P* < 0.05). GR, MR, 11βHSD1, and 11βHSD2 showed positive age-related regression in the PVN (R values were 0.78, 0.79, 0.76, 0.73, *P* < 0.05 respectively). CRH and p-GR were not correlated with ages (*P* > 0.05). The expression of H6PD tended to correlate positively with age in the PVN of hypothalamus (R = 0.49, *P* = 0.08). There was some staining of AVP in capillary endothelia in the PVN of the younger animals.

### Potential local feedback and cortisol generation within the PVN

Correlations were calculated for those proteins that showed an age-related change. 11βHSD1 correlated negatively with AVP (R = −0.51, *P* = 0.03), and positively with GR (R = 0.51, *P* = < 0.03), MR (R = 0.67, *P* < 0.01), and 11βHSD2 (R = 0.72, *P* < 0.01).

### Abundance and age-related correlations of PVN immunoreactive pituitary proteins

**Figure 4:**
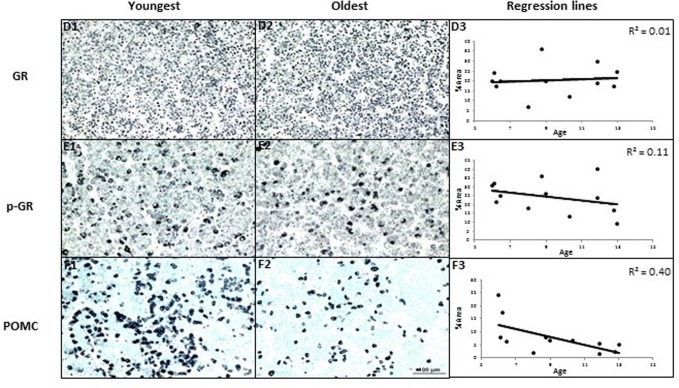
The effects of age on area% positive staining of proteins in pituitary of female baboons (*P. hamadryas*) POMC expression showed negative age-related linear regression in anterior pituitary (n = 12, R = −0.63, P < 0.05). GR and p-GR were not correlated with age. Scale bar (100µm) applies to all micrographs.

Immunoreactive GR, p-GR, and POMC were detected in the pituitary. Pituitary immunoreactive POMC correlated negatively with age. GR and p-GR expression showed no correlations with age in the pituitary (Fig. 4).

## DISCUSSION

Several stressors, defined as challenges that disrupt the body's internal homeostatic balance, lead to premature aging [14]. HPAA activity is central to mediation of effects of stressors on organs throughout the body. The HPAA is regulated by CRH and AVP; both are secreted by the hypothalamic PVN and both stimulate conversion of POMC to adrenocorticotropin (ACTH) in the pituitary. Pituitary ACTH is then secreted into circulation and stimulates the release of adrenal steroids, including cortisol and dehydroepiandrosterone sulfate (DHEAS). Cortisol is the active glucocorticoid in both nonhuman primates and humans and acts on peripheral tissues through two receptors: the glucocorticoid receptor (GR) and the mineralocorticoid receptor (MR). Cortisol acts via these two receptors as a negative feedback brake on the hypothalamus. GR is activated by phosphorylation to form phospho-GR (p-GR) [15]. GR and p-GR are widely expressed throughout the central nervous system, especially in hypothalamic neurons [16, 17], where they mediate the feedback that regulates HPAA responsiveness to stress [18, 19].

Glucocorticoids are pleiotropic regulators that control multiple critical life-course age-related functions in many physiological systems [6, 20]. Overall frailty and weakness similar to that observed with aging occur in the presence of both cortisol excess (Cushing's disease) [21] and cortisol deficiency (Addison's disease) [22]. An extensive literature search reveals considerable variability in the profile of normative changes in human and animal circulating glucocorticoid (corticosterone in most rodents and cortisol in most precocial species) concentrations across the life-course [7]. Existing reviews on glucocorticoids’ roles in determining life-course health suggest a potential key role in aging [23, 24]. However, conflicting information exists as to whether glucocorticoid basal and/or stimulated levels increase or decrease with age. A major problem in reaching conclusions about life-course changes in nonhuman primate glucocorticoids is that many well-conducted studies in primate colonies were designed to obtain data to answer questions unrelated to aging effects and changes related to aging of the subjects included in the study were only subsequently assessed as an additional goal. One excellent study entitled “Plasma cortisol responses to stress in lactating and non-lactating female rhesus macaques” obtained samples from 53 lactating and non-lactating rhesus monkeys that lived in six different naturally formed social groups [25]. This study in female rhesus macaques of which approximately half were lactating and half non-pregnant and non-lactating demonstrated a fall in cortisol across the life-course but the fall was not significant (p < 0.09). To conduct this study free ranging animals were assembled and moved to indoor cages for blood sampling after overnight housing. Lactating mothers showed significantly higher plasma cortisol than non-lactating females [25]. Presence of lactation or not predicted cortisol better than age or dominance rank. Thus it is relevant to our findings reported here that in a study designed for purposes other than obtaining age related data and with the major extra factor of presence or absence of lactation, that there should even be a close approximation to a correlation with age. The stress involved with movement on the day prior to the study may also have had an effect that differed in young and old animals since investigators noted, *“Capture, handling, and individual housing in a cage are powerful psychological stressors for free-ranging primates*” and state, “*We suggest that the higher plasma cortisol levels exhibited by lactating females reflect greater responsiveness to stress associated with perception of risks to infants*” [25].

The strongest support for our observation that given the correct conditions in animals of relatively similar phenotype within one colony, cortisol falls across the life-course, comes from the recently published study from the Oklahoma primate center [8]. There is a remarkable similarity in these two studies in the rate of fall of cortisol 24.7 ng.ml ^−1^.y^—1^ in our study and 23.7 ng.ml ^−1^.y^−-1^in the Oklahoma study (Fig. 2).

Several possibilities could be proposed for the differences in absolute cortisol values between our studies and those in the Oklahoma primate center– among them are assay differences, prior life experiences including developmental programming, housing conditions, environmentally induced phenotype differences, and past health history of the subjects studied. These possibilities remain to be evaluated but the remarkable similarity in the linear decrease in cortisol with age remains.

Homogeneous social groupings and stable conditions are needed to establish intrinsic normative changes. Another factor that must be considered is that almost all studies have evaluated life-course changes using comparison between data obtained in limited groups of ages and not across major sections of the life-course (for review see [6]). To avoid these limitations in our rat study we investigated corticosterone at five time-points across the life-course in a well-characterized homogeneous rat colony with a life span around 850 days. We determined the profile of circulating corticosterone in the early active period in the rat activity rhythm (early night) from weaning (21 PND) to old age (850 PND). While not as powerful as single linear regression throughout the period of study in this carefully controlled situation we observed a decline in corticosterone concentrations in the second half of life, with higher levels in females. The timing of the decrease with aging was similar in both sexes [6].

We and others have used the tether system in nonhuman primates to permit frequent sampling without the stress of acute multiple venepuncture [26–28]. These systems are powerful approaches to studying physiology of many systems including neuroendocrine systems and have been extensively employed by others and ourselves. One tethered study in rhesus monkeys reports a rise in cortisol across the life span – a result very different from our own presented here and the Oklahoma findings [8], which agree closely. We do not favour the explanation that this difference is a species difference since as comparative physiologists we hold to the idea that similar systems will generally operate across primates – both nonhuman and human. Thus further studies need to be conducted to determine the effects of tethering and housing in single cages to determine whether they alter cortisol levels and trajectories of aging. It is of interest that the three studies, two baboon and one rhesus, that did not use tether all show a fall – albeit with a p value of only 0.09 in the rhesus study.

Several studies have now demonstrated local production of cortisol within a variety of tissues by the 11βHSD system [12, 29] and brain [13]. In the brain this local production has been shown to affect phenotype [13]. The strong negative correlation between 11βHSD1 and its cofactor H6PD, but not 11βHSD2, with AVP may indicate a hypothalamic role in local cortisol generation in driving the age-related fall in AVP and hence cortisol. Interestingly 11βHSD1 also correlated positively with GR and MR. Local generation of cortisol is negatively regulated by 11βHSD2 and it is thus of interest that 11βHSD2 protein abundance is also increased and also correlates positively with 11βHSD1, H6PD, GR, and MR – possibly as a compensatory mechanism. Small amounts of AVP staining in the capillary endothelium in Figure 3 most likely reflects the fact that AVP secreted by the paraventricular nuclear cells has to pass through the capillary endothelium to enter the blood and circulate to its many targets, including the kidney. In keeping with this, the endothelial staining is greater in A1, the example of a younger baboon, compared to A2, an older example, since the younger baboons are producing more AVP.

Systemically administered glucocorticoids inhibit AVP and CRH [30]. To our knowledge this study is the first to investigate a potential role of age-related alterations in the drive (AVP, CRH, and POMC) as well as negative feedback (GR and MR) in the PVN and pituitary. The observations that GR and MR increased with age as well as 11βHSD1 are all in keeping with our hypothesis that increased central negative feedback occurred with aging.

### Mechanistic considerations of the cause and consequence of the age-related fall in cortisol

Our data identify increased negative feedback and local PVN cortisol production as mechanisms responsible for the decreased PVN drive to HPAA with aging. In the future to determine the functional role of the fall in cortisol on life-course function, studies need to be designed to prevent the fall and observe the consequences. Such a cortisol replacement study will be aided by the remarkable similarity in the rate of fall in the study reported here in our study - 24 ng.ml ^−1^.y^−1^ and that in the Oklahoma baboon colony - 23.7 ng.ml ^−1^.y^−-1^. It is necessary to consider three potential differing functional significances for the fall. First the fall in cortisol may cause aging changes in which case the replacement should slow the rate of aging and extend the health span. Alternatively since cortisol does damage tissue and at high levels would be expected to increase the rate of aging, the fall in cortisol may be a physiological system that slows the rate of aging. Of these two possibilities the protective effect of reducing circulating glucocorticoids is supported by studies in a rat model in which dietary restriction is shown to have neuroprotective actions in the presence of an increase in corticosterone. When the dietary restriction-induced increase in corticosterone is prevented by adrenalectomy, the neuroprotective effects are increased [31]. These findings support the view that dietary restriction-induced increase in levels of corticosterone has potentially negative effects that counteract the benefits of dietary restriction.

A third possibility is that the fall is an epiphenomenon unrelated to the aging process. Given the important ubiquitous effects of the HPAA on physiological processes it will be important to conduct experiments that distinguish between these interesting alternatives.

Cortisol secreted by the adrenal as well as that produced locally likely has concentration and age dependent effects that may result in bimodal or U-shaped patterns of outcomes in relation to aging. Similar potentially opposing effects of the fall in insulin-like growth factor-1 (IGF1) that occurs across the life-course may also influence its effects on the life span. IGF1 has been shown to have actions that promote cell survival within the vascular system as well as reduce cellular life span [32]. In addition high levels of IGF1 promote several forms of neoplasia that would shorten life [33]. Another parallel between IGF and cortisol in aging is the potential role of locally produced IGF1. In a study on S1S2 transgenic mice overexpression initially had beneficial effects on systolic function but these reversed with time [34]. Clearly in this type of situation life-course change in both local and systemic hormone function must be evaluated. A more complete awareness of the relationship between glucocorticoids and the aging process across species will aid in translation to achieve better understanding of the role of HPAA in human aging and potential interventions to increase health span.

## MATERIALS AND METHODS

### Animal care and maintenance

All procedures were approved by the Texas Biomedical Research Institute (TBRI) Animal Care and Use Committee and conducted in facilities approved by the Association for Assessment and Accreditation of Laboratory Animal Care. The baboons (*Papio hamadryas*) were housed in 20 × 20 × 15 foot metal and concrete group cages at the Southwest National Primate Research Center, at TBRI, in San Antonio, Texas. Diet was normal Primate Center laboratory chow (12% energy from fat with 0.29% glucose and 0.32% fructose and energy content of 3.07 kcal/g protein) Purina Monkey Diet 5038 (Purina, St Louis, MO, USA) *ad libitum*, a complete life-cycle diet for Old World primates. Water was continuously available in each feeding cage. Animal food consumption, weights, and health status were recorded daily. All animals were given a full veterinary examination prior to recruitment to the study and no obvious cause of ill health was observed.

### Hormone quantification

Baboons were trained and accustomed to run from their group cages through a chute into individual cages in sight of all the other baboons normally in their group cage for many different types of procedures, including rewards, and thus were well adapted to the procedure. Following an overnight fast, once in the individual cage ketamine was administered intramuscularly (IM) at 10 mg/kg between 8:00 AM – 9:00 AM followed in less than 5 minutes by removal of a femoral venous blood sample. Time of day of blood sampling was controlled to minimize variation from circadian rhythms. Serum cortisol concentration was determined by chemiluminescent immunoassay on an Immulite 1000 analyzer with kits from Siemens Healthcare Diagnostics (Flanders, New Jersey). Pooled serum was first tested to validate the performance of this system for baboon samples. Assay precision was determined by testing pooled samples using 5 replicates in each assay. These assays were repeated at 2 dilutions to assess linearity of the results. All test samples were run at dilutions estimated to achieve values in the middle of the assay calibration range. The intraassay and interassay coefficients of variation for cortisol were 5.6 and 8.4, respectively.

### Lack of effect of ketamine on peripheral cortisol levels within 10 minutes of administration

Baboons were acclimatized for six weeks to wearing a jacket and tether system that was connected to a swivel apparatus allowing free movement in individual cages without any exposure to pharmacological agents [35]. After acclimation, the femoral artery and vein in six male and six female baboons were catheterized under general anesthesia. Following full recovery from anesthesia baboons were reconnected to the tether system. We demonstrated that the tranquilizing dose of ketamine used for immobilization (10 mg/kg IM) does not raise circulating cortisol in the 10 min maximum time needed to take a blood sample. At least a week after surgery for placement of the vascular catheters, a baseline blood sample was taken through the venous catheter while the baboon was fully conscious and free moving without administration of any agents in the last 24 h. Ketamine (10 mg/kg IM) was then administered and femoral venous samples taken at 5 and 10 min after ketamine.

### Necropsy

For necropsy baboons were pre-medicated with ketamine hydrochloride (10 mg/kg IM) and anesthetized using isoflurane (2%) as previously described [36]. Baboons were exsanguinated while still under general anesthesia as approved by the American Veterinary Medical Association. Following cardiac asystole, brain tissue was collected and hypothalamus and pituitary were separated rapidly.

### Histological processing and validation of antibodies

Hypothalami and pituitary glands from females aged 6-13 years were fixed for 24 hours with 4% paraformaldehyde solution, dehydrated, and blocked in paraffin. Adjacent 5 µm coronal paraffin slices of the hypothalamic PVN were immunostained for AVP, CRH, MR, GR, p-GR, 11βHSD1, 11βHSD2, and H6PD, as well as pituitary GR, p-GR, and POMC. Source and final concentrations of the primary antibodies for the proteins together with validation procedures – western blot or preabsorption with immunizing antigen – are presented in Table 1. Immunohistochemical methods have been described in detail [37]. The final optimal concentration of the primary antibodies for the proteins was determined using our published methods [38]. The parvo and magnocellular areas of the PVN were studied together. As in other published studies of PVN secretagogues we did not separate parvocellular and magnocellular neurons [39]. Images were obtained with a SPOT RT3 cooled color digital camera (2650×1920 pixels, Diagnostic Instruments, McHenry, IL, USA) mounted on a Nikon E600 microscope (Nikon, Inc., Melville, NY, USA) as previously reported [40].

**Table 1:** Antibody antigens, final dilutions, and sources used for immunohistochemical staining of baboon (*P. hamadryas*) brain tissue

Antigen	Company	Cat#	Validation	Tissue staining	Concentration
AVP	Chemicon-Millipore	AB-1565	WB^1^	Hypothalamus	1:20k
CRH	Santa Cruz	sc-10718	WB	Hypothalamus	1:150
GR	Michael Garabedian	NYU	Pre-absorbed^2^	Hypothalamus	1:1500
GR	Santa Cruz	sc-8992	WB	Pituitary	1:100
p-GR	Michael Garabedian	NYU	Pre-absorbed	Hypothalamus	1:500
p-GR	Michael Garabedian	NYU	Pre-absorbed	Pituitary	1:1k
MR	Santa Cruz	sc-11412	WB	Hypothalamus	1:500
11βHSD1	Santa Cruz	sc-20175	WB	Hypothalamus	1:200
11βHSD2	Santa Cruz	sc-20176	WB	Hypothalamus	1:200
H6PD	Santa Cruz	Sc-67394	WB	Hypothalamus	1:200
POMC	Phoenix Pharmaceuticals	H-029-30	WB	Pituitary	1:100k

1 Western blot

2 Tested by pre-absorption of the antigen

### Statistical analysis

To determine the effects of age on all proteins observed, each was regressed against age, using a simple linear regression model in all cases for consistency. Significance was set at *P* < 0.05. In the few cases where a nonlinear (i.e., exponential) model seemed attractive, improvements by so doing were insufficiently compelling.

## Abbreviations

11βHSD: 11beta hydroxysteroid dehydrogenase Type 1
11βHSD2: 11beta hydroxysteroid dehydrogenase Type 2
AVP: Arginine vasopressin
CRH: Corticotropin-releasing hormone
GR: Glucocorticoid receptor
H6PD: Hexose-6-phosphate dehydrogenase
HPAA: Hypothalamo-pituitary-adrenal axis
MR: Mineralo-corticoid receptor
p-GR: Phospho-glucocorticoid receptor
PND: Postnatal days
POMC: Pro-opiomelanocortin
PVN: Paraventricular nucleus
